# A deep learning approach to bilingual lexicon induction in the biomedical domain

**DOI:** 10.1186/s12859-018-2245-8

**Published:** 2018-07-09

**Authors:** Geert Heyman, Ivan Vulić, Marie-Francine Moens

**Affiliations:** 1 LIIR, Department of Computer Science, Celestijnenlaan 200A, Leuven, Belgium; 2 Language Technology Lab, DTAL, University of Cambridge, 9 West Road, Cambridge, UK

**Keywords:** Bilingual lexicon induction, Medical terminology, Representation learning, Biomedical text mining

## Abstract

**Background:**

Bilingual lexicon induction (BLI) is an important task in the biomedical domain as translation resources are usually available for general language usage, but are often lacking in domain-specific settings. In this article we consider BLI as a classification problem and train a neural network composed of a combination of recurrent long short-term memory and deep feed-forward networks in order to obtain word-level and character-level representations.

**Results:**

The results show that the word-level and character-level representations each improve state-of-the-art results for BLI and biomedical translation mining. The best results are obtained by exploiting the synergy between these word-level and character-level representations in the classification model. We evaluate the models both quantitatively and qualitatively.

**Conclusions:**

Translation of domain-specific biomedical terminology benefits from the character-level representations compared to relying solely on word-level representations. It is beneficial to take a deep learning approach and learn character-level representations rather than relying on handcrafted representations that are typically used. Our combined model captures the semantics at the word level while also taking into account that specialized terminology often originates from a common root form (e.g., from Greek or Latin).

## Introduction

As a result of the steadily growing process of globalization, there is a pressing need to keep pace with the challenges of multilingual international communication. New technical specialized terms such as biomedical terms are generated on almost a daily basis, and they in turn require adequate translations across a plethora of different languages. Even in local medical practices we witness a rising demand for translation of clinical reports or medical histories [1]. In addition, the most comprehensive specialized biomedical lexicons in the English language such as the Unified Medical Language System (UMLS) thesaurus lack translations into other languages for many of the terms^1^.

**Fig. 1:**
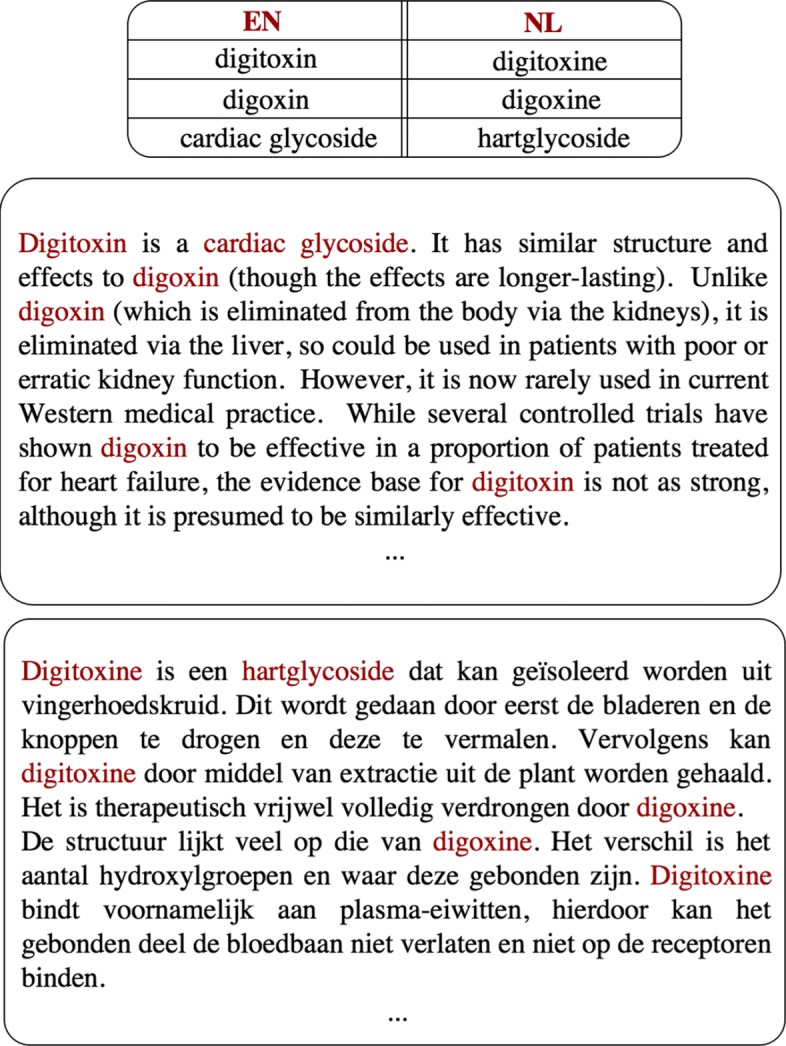
Comparable corpora. Excerpts of the English-Dutch comparable corpus in the biomedical domain that we used in the experiments with a few domain-specific translations indicated in red

Translation dictionaries and thesauri are available for most language pairs, but they typically do not cover domain-specific terminology such as biomedical terms. Building bilingual lexicons that contain such terminology by hand is time-consuming and requires trained experts. As a consequence, we observe interest in automatically learning the translation of terminology from a corpus of domain-specific bilingual texts [2]. What is more, in specialized domains such as biomedicine, parallel corpora are often not readily available: therefore, translations are mined from non-parallel comparable bilingual corpora [3, 4]. In a parallel corpus every sentence in the source language is linked to a translation of that sentence in the target language, while in a comparable corpus, the texts in source and target language contain similar content, but are not exact translations of each other: as an illustration, Fig. 1 shows a fragment of the biomedical comparable corpus we used in our experiments. In this article we propose a deep learning approach to bilingual lexicon induction (BLI) from a comparable biomedical corpus.

Neural network based deep learning models [5] have become popular in natural language processing tasks. One motivation is to ease feature engineering by making it more automatic or by learning end-to-end. In natural language processing it is difficult to hand-craft good lexical and morpho-syntactic features, which often results in complex feature extraction pipelines. Deep learning models have also made their breakthrough in machine translation [6, 7], hence our interest in using deep learning models for the BLI task. Neural networks are typically trained using a large collection of texts to learn distributed representations that capture the contexts of a word. In these models, a word can be represented as a low-dimensional vector (often referred to as a word embedding) which embeds the contextual knowledge and encodes semantic and syntactic properties of words stemming from the contextual distributional knowledge [8].

Lately, we also witness an increased interest in learning character representations, which better capture morpho-syntactic properties and complexities of a language. What is more, the character-level information seems to be especially important for translation mining in specialized domains such as biomedicine as such terms often share common roots from Greek and Latin (see Fig. 1), or relate to similar abbreviations and acronyms.

Following these assumptions, in this article we propose a novel method for mining translations of biomedical terminology: the method integrates character-level and word-level representations to induce an improved bilingual biomedical lexicon.

## Background and contributions

**BLI in the biomedical domain** Bilingual lexicon induction (BLI) is the task of inducing word translations from raw textual corpora across different languages. Many information retrieval and natural language processing tasks benefit from automatically induced bilingual lexicons, including multilingual terminology extraction [2], cross-lingual information retrieval [9–12], statistical machine translation [13, 14], or cross-lingual entity linking [15]. Most existing works in the biomedical domain have focused on terminology extraction from biomedical documents but not on terminology translation. For instance, [16] use a combination of off-the-shelf components for multilingual terminology extraction but do not focus on learning terminology translations. The OntoLearn system extracts terminology from a corpus of domain texts and then filters the terminology using natural language processing and statistical techniques, including the use of lexical resources such as WordNet to segregate domain-general and domain-specific terminology [17]. The use of word embeddings for the extraction of domain-specific synonyms was probed by Wang et al. [18].

Other works have focused on machine translation of biomedical documents. For instance, [19] compared the performance of neural-based machine translation with classical statistical machine translation when trained on European Medicines Agency leaflet texts, but did not focus on learning translations of medical terminology. Recently, [20] explored the use of existing word-based automated translators, such as Google Translate and Microsoft Translator, to translate English UMLS terms into French and to expand the French terminology, but do not construct a novel methodology based on character-level representations as we propose in this paper. Most closely related to our work is perhaps [21], where a label propagation algorithm was used to find terminology translations in an English-Chinese comparable corpus of electronic medical records. Different from the work presented in this paper, they relied on traditional co-occurrence counts to induce translations and did not incorporate information on the character level.

**BLI and word-level information** Traditional bilingual lexicon induction approaches aim to derive cross-lingual word similarity from either context vectors, or bilingual word embeddings. The context vector of a word can be constructed from (1) weighted co-occurrence counts ([2, 22–27], inter alia), or (2) monolingual similarities [28–31] with other words.

The most recent BLI models significantly outperform traditional context vector-based baselines using bilingual word embeddings (BWE) [24, 32, 33]. All BWE models learn a distributed representation for each word in the source- and target-language vocabularies as a low-dimensional, dense, real-valued vector. These properties stand in contrast to traditional count-based representations, which are high-dimensional and sparse. The words from both languages are represented in the same vector space by using some form of bilingual supervision (e.g., word-, sentence- or document-level alignments) ([14, 34–41], inter alia)^2^. In this cross-lingual space, similar words, regardless of the actual language, obtain similar representations.

To compute the semantic similarity between any two words, a similarity function, for instance cosine, is applied on their bilingual representations. The target language word with the highest similarity score to a given source language word is considered the correct translation for that source language word. For the experiments in this paper, we use two BWE models that have obtained strong BLI performance using a small set of translation pairs [34], or document alignments [40] as their bilingual signals.

The literature has investigated other types of word-level translation features such as raw word frequencies, word burstiness, and temporal word variations [44]. The architecture we propose enables incorporating these additional word-level signals. However, as this is not the main focus of our paper, it is left for future work.

**BLI and character-level information** Etymologically similar languages with shared roots such as English-French or English-German often contain word translation pairs with shared character-level features and regularities (e.g., *accomplir:accomplish*, *inverse:inverse*, *Fisch:fish*). This orthographic evidence comes to the fore especially in domains such as legal domain or biomedicine. In such expert domains, words sharing their roots, typically from Greek and Latin, as well as acronyms and abbreviations are abundant. For instance, the following pairs are English-Dutch translation pairs in the biomedical domain: *angiography:angiografie*, *intracranial:intracranieel*, *cell membrane:celmembraan*, or *epithelium:epitheel*. As already suggested in prior work, such character-level evidence often serves as a strong translation signal [45, 46]. BLI typically exploits this through string distance metrics: for instance, Longest Common Subsequence Ratio (LCSR) has been used [28, 47], as well as edit distance [45, 48]. What is more, these metrics are not limited to languages with the same script: their generalization to languages with different writing systems has been introduced by Irvine and Callison-Burch [44]. Their key idea is to calculate normalized edit distance only after transliterating words to the Latin script.

As mentioned, previous work on character-level information for BLI has already indicated that character-level features often signal strong translation links between similarly spelled words. However, to the best of our knowledge our work is the first which learns bilingual character-level representations from the data in an automatic fashion. These representations are then used as one important source of translation knowledge in our novel BLI framework. We believe that character-level bilingual representations are well suited to model biomedical terminology in bilingual settings, where words with common Latin or Greek roots are typically encountered [49]. In contrast to prior work, which typically resorts to simple string similarity metrics (e.g., edit distance [50]), we demonstrate that one can induce bilingual character-level representations from the data using state-of-the-art neural networks.

**Framing BLI as a classification task** Bilingual lexicon induction may be framed as a discriminative classification problem, as recently proposed by Irvine and Callison-Burch [44]. In their work, a linear classifier is trained which blends translation signals as similarity scores from heterogeneous sources. For instance, they combine translation indicators such as normalized edit distance, word burstiness, geospatial information, and temporal word variation. The classifier is trained using a set of known translation pairs (i.e., training pairs). This combination of translation signals in the supervised setting achieves better BLI results than a model which combines signals by aggregating mean reciprocal ranks for each translation signal in an unsupervised setting. Their model also outperforms a well-known BLI model based on matching canonical correlation analysis from Haghighi et al. [45]. One important drawback of Irvine and Callison-Burch’s approach concerns the actual fusion of heterogeneous translation signals: they are transformed to a similarity score and weighted independently. Our classification approach, on the other hand, detects word translation pairs by learning to combine word-level and character-level signals in the joint training phase.

**Contributions** The main contribution of this work is a *novel bilingual lexicon induction framework*. It combines character-level and word-level representations, where both are automatically extracted from the data, within a discriminative classification framework^3^. Similarly to a variety of bilingual embedding models [52], our model requires translation pairs as a bilingual signal for training. However, we show that word-level and character-level translation evidence can be effectively combined within a classification framework based on deep neural nets. Our state-of-the-art methodology yields strong BLI results in the biomedical domain. We show that incomplete translation lists (e.g., from general translation resources) may be used to mine additional domain-specific translation pairs in specialized areas such as biomedicine, where seed general translation resources are unable to cover all expert terminology. In sum, the list of contributions is as follows.

First, we show that bilingual character-level representations may be induced using an RNN model. These representations serve as better character-level translation signals than previously used string distance metrics. Second, we demonstrate the usefulness of framing term translation mining and bilingual lexicon induction as a discriminative classification task. Using word embeddings as classification features leads to improved BLI performance when compared to standard BLI approaches based on word embeddings, which depend on direct similarity scores in a cross-lingual embedding space. Third, we blend character-level and word-level translation signals within our novel deep neural network architecture. The combination of translation clues improves translation mining of biomedical terms and yields better performance than “single-component” BLI classification models based on only one set of features (i.e., character-level or word-level). Finally, we show that the proposed framework is well suited for finding *multi-word translations pairs* which are also frequently encountered in biomedical texts across different languages.

## Methods

As mentioned, we frame BLI as a classification problem as it supports an elegant combination of word-level and character-level representations. In this section, we have taken over parts of the previously published work [51] that this paper expands.

Let *V*^*S*^ and *V*^*T*^ denote the source and target vocabularies respectively, and *C*^*S*^ and *C*^*T*^ denote the sets of all unique source and target characters. The vocabularies contain all unique words in the corpus as well as phrases (e.g., *autoimmune disease*) that are automatically extracted from the corpus. We use *p* to denote a word or a phrase. The goal is to learn a function *g*:*X*→*Y*, where the input space *X* consists of all candidate translation pairs *V*^*S*^×*V*^*T*^ and the output space *Y* is {−1,+1}. We define *g* as:

$$\begin{array}{*{20}l} g\left(p^{S}, p^{T}\right) = \left\{ \begin{array}{ll} +1 &\text{, if } f\left(p^{S}, p^{T}\right) > t \\ -1 &\text{, otherwise} \end{array}\right. \end{array} $$

Here, *f* is a function realized by a neural network that produces a classification score between 0 and 1; *t* is a threshold tuned on a validation set. When the neural network is confident that *p*^*S*^ and *p*^*T*^ are translations, *f*(*p*^*S*^,*p*^*T*^) will be close to 1. The motivation for placing a threshold *t* on the output of *f* is twofold. First, it allows balancing between recall and precision. Second, the threshold naturally accounts for the fact that words might have multiple translations: if two target language words/phrases $p_{1}^{T}$ and $p_{2}^{T}$ both have high scores when paired with *p*^*S*^, both may be considered translations of *p*^*S*^.

Note that the classification approach is methodologically different from the classical *similarity-driven* approach to BLI based on a similarity score in the shared bilingual vector space. Cross-lingual similarity between words *p*^*S*^ and *p*^*T*^ is computed as $SF\left (r_{p}^{S},r_{p}^{T}\right)$, where $r_{p}^{S}$ and $r_{p}^{T}$ are word/phrase representations in the shared space, and *SF* denotes a similarity function operating in the space (cosine similarity is typically used). A target language term *p*^*T*^ with the highest similarity score $\arg \max _{p^{T}} SF\left (r_{p}^{S},r_{p}^{T}\right)$ is then taken as the correct translation of a source language word *p*^*S*^.

Since neural network parameters are trained using a set of translation pairs *D*_*lex*_, *f* in our classification approach can be interpreted as an automatically trained similarity function. For each positive training translation pair <*p*^*S*^,*p*^*T*^>, we create 2*N*_*s*_*noise* or *negative* training pairs. These negative samples are generated by randomly sampling *N*_*s*_ target language words/phrases $p_{neg,S,i}^{T}$, *i*=1,…,*N*_*s*_ from *V*^*T*^ and pairing them with the source language word/phrase *p*^*S*^ from the true translation pair <*p*^*S*^,*p*^*T*^>.^4^ Similarly, we randomly sample *N*_*s*_ source language words/phrases $p_{neg,T,i}^{S}$ and pair them with *p*^*T*^ to serve as negative samples. We then train the network by minimizing the cross-entropy loss, a commonly used loss function for classification that optimizes the likelihood of the training data. The loss function is expressed by Eq. 1, where *D*_*neg*_ denotes the set of negative examples used during training, and where *y* denotes the binary label for <*p*^*S*^,*p*^*T*^> (1 for valid translation pairs, 0 otherwise).

1 $$\begin{array}{*{20}l} \mathcal{L}_{ce} =& \sum\limits_{<p^{S}, p^{T}>\in D_{lex} \cup D_{neg}} -y\log\left(f\left(p^{S}, p^{T}\right)\right) \\&- (1 - y)\log\left(1 - f\left(p^{S}, p^{T}\right)\right)  \end{array} $$

We further explain the architecture of the neural network, the approach to construct vocabularies of words and phrases and the strategy to identify candidate translations during prediction. Four key components may be distinguished: (1) the input layer; (2) the character-level encoder; (3) the word-level encoder; and (4) a feed-forward network that combines the output representations from the two encoders into the final classification score.

### Input layer

The goal is to exploit the knowledge encoded in both the word and character levels. Therefore, the raw input representation of a word/phrase *p*∈*V*^*S*^ of character length *M* consists of (1) its *one-hot* encoding on the word level, labeled $x^{S}_{p}$; and (2) a sequence of *M* one-hot encoded vectors $x^{S}_{c0},..,x^{S}_{ci},..x^{S}_{cM}$ on the character level, representing the character sequence of the word. $x^{S}_{p}$ is thus a |*V*^*S*^|-dimensional word vector with all zero entries except for the dimension that corresponds to the position of the word/phrase in the vocabulary. $x^{S}_{ci}$ is a |*C*^*S*^|-dimensional character vector with all zero entries except for the dimension that corresponds to the position of the character in the character vocabulary *C*^*S*^.

### Character-level encoder

**Fig. 2:**
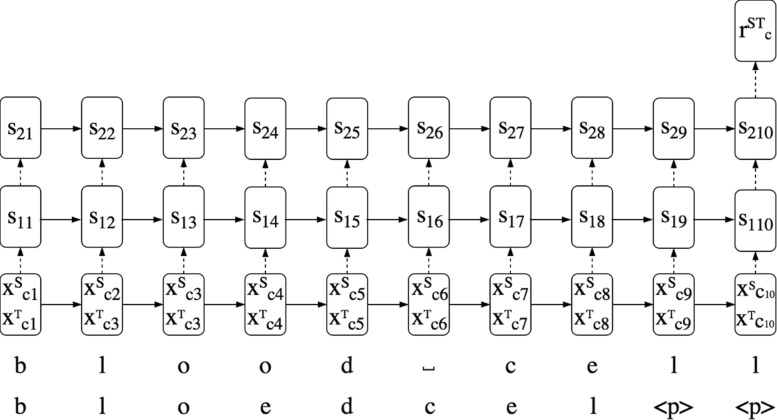
Character-level encoder. An illustration of the character-level LSTM encoder architecture using the example EN-NL translation pair <*blood cell, bloedcel* >

To encode a pair of character sequences $x^{S}_{c0},..,x^{S}_{ci},..x^{S}_{cn}$, $x^{T}_{c0},..,x^{T}_{ci},..x^{T}_{cm}$ we use a two-layer long short-term memory (LSTM) recurrent neural network (RNN) [53] as illustrated in Fig. 2. At position *i* in the sequence, we feed the concatenation of the *i*^*th*^ character of the source language and target language word/phrase from a training pair to the LSTM network. The space character in phrases is threated like any other character. The characters are represented by their one-hot encoding. To deal with the possible difference in word/phrase length, we append special padding characters at the end of the shorter word/phrase (see Fig. 2). *s*_1*i*_, and *s*_2*i*_ denote the states of the first and second layer of the LSTM. We found that a two-layer LSTM performed better than a shallow LSTM. The output at the final state *s*_2*N*_ is the character-level representation $r^{ST}_{c}$. We apply dropout regularization [54] with a keep probability of 0.5 on the output connections of the LSTM (see the dotted lines in Fig. 2). We will further refer to this architecture as CHARPAIRS^5^.

### Word-level encoder

We define the word-level representation of a pair <*p*^*S*^,*p*^*T*^> simply as the concatenation of the embeddings for *p*^*S*^ and *p*^*T*^:

2 $$\begin{array}{*{20}l} r^{ST}_{p} = W^{S} \cdot x^{S}_{p} \hspace{3mm} \| \hspace{3mm} W^{T} \cdot x^{T}_{p} \end{array} $$

Here, $r^{ST}_{p}$ is the representation of the word/phrase pair, and *W*^*S*^, *W*^*T*^ are word embedding matrices looked up using one-hot vectors $x^{S}_{p}$ and $x^{T}_{p}$. In our experiments, *W*^*S*^ and *W*^*T*^ are obtained in advance using any state-of-the-art word embedding model, e.g., [34, 40] and are then kept *fixed* when minimizing the loss from Eq. 1.

To test the generality of our approach, we experiment with two well-known embedding models: (1) the model from Mikolov et al. [34], which trains monolingual embeddings using skip-gram with negative sampling (SGNS) [8]; and (2) the model of Vulić and Moens [40] which learns word-level bilingual embeddings from document-aligned comparable data (BWESG). For both models, the top layers of our proposed classification network should learn to relate the word-level features stemming from these word embeddings using a set of annotated translation pairs.

### Combination: feed-forward network

To combine these word-level and character-level representations we use a fully connected feed-forward neural network *r*_*h*_ on top of the concatenation of $r^{ST}_{p}$ and $r^{ST}_{c}$ which is fed as input to the network:

3 $$\begin{array}{*{20}l} & r_{h_{0}} = r^{ST}_{p} \| r^{ST}_{c}  \end{array} $$

4 $$\begin{array}{*{20}l} & r_{h_{i}} = \sigma\left(W_{h_{i}} \cdot r_{h_{i-1}} + b_{h_{i}}\right) \end{array} $$

5 $$\begin{array}{*{20}l} & score = \sigma\left(W_{o} \cdot r_{h_{H}} + b_{o}\right) \end{array} $$

**Fig. 3:**
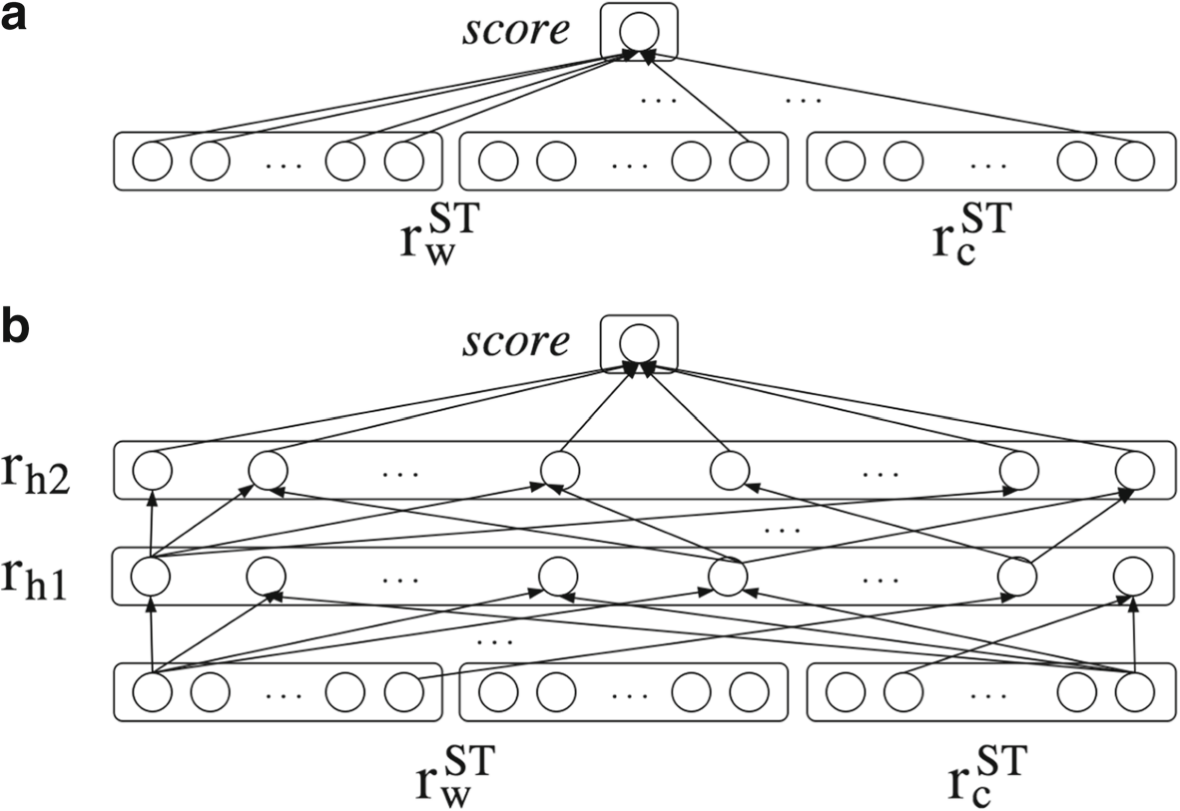
Classification component. Illustrations of the classification component with feed-forward networks of different depths. **a**: *H*=0. **b**: *H*=2 (our model). All layers are fully connected. This figure is taken from [51]

*σ* denotes the sigmoid function and *H* denotes the number of layers between the representation layer and the output layer. In the simplest architecture, *H* is set to 0 and the word-pair representation $r_{h_{0}}$ is directly connected to the output layer (see Fig. 3a, Figure taken from [51]). In this setting each dimension from the concatenated representation is weighted independently. This is undesirable as it prohibits learning relationship between the different representations. On the word level, for instance, it is obvious that the classifier needs to combine the embeddings of the source and target word to make an informed decision and not merely calculate a weighted sum of them. Therefore, we opt for an architecture with hidden layers instead (see Fig. 3b). Unless stated otherwise, we use two hidden layers, while in Experiment V of the “Results and discussion” section we further analyze the influence of parameter *H*.

### Constructing the vocabularies

The vocabularies are the union of all words that occur at least five times in the corpus and phrases that are automatically extracted from it. We opt for the phrase extraction method proposed in [8]^6^. The method iteratively extracts phrases for bigrams, trigrams, etc. First, every bigram is assigned a score using Eq. 6. Bigrams with a score greater than a given threshold are added to the vocabulary as phrases. In subsequent iterations, extracted phrases are treated as if they were a single token and the same process is repeated. The threshold and the value for *δ* are set so that we maximize the recall of the phrases in our training set. We performed 4 iterations in total, resulting in N-grams up to a length of 5.

When learning the word-level representations phrases are treated as a single token (following Mikolov et al. [8]). Therefore, we do not add words that only occur as part of a phrase separately to the the vocabulary, because no word representation is learned for these words. E.g., for our dataset *“York”* is not included in the vocabulary as it always occurs as part of the phrase *“New York”*.

6 $$\begin{array}{*{20}l} & score(w_{i}, w_{j}) = \frac{Count(w_{i}, w_{j}) - \delta}{Count(w_{i}) \cdot Count(w_{j})} \cdot |V|,  \end{array} $$

*Count*(*w*_*i*_,*w*_*j*_) is the frequency of the bigram *w*_*i*_ *w*_*j*_, *Count(w)* is the frequency of *w*, |*V*| is the size of the vocabulary, and *δ* is a discounting coefficient that prevents that too many phrases consist of very infrequent words.

### Candidate generation

To identify which word pairs are translations, one could enumerate all translation pairs and feed them to the classifier *g*. The time complexity of this brute-force approach is *O*(|*V*^*S*^|×|*V*^*T*^|) times the complexity of *g*. For large vocabularies this can be a prohibitively expensive procedure. Therefore, we have resorted to a heuristic which uses a noisy classifier: it generates 2*N*_*c*_<<|*V*^*T*^| translation candidates for each source language word/phrase *p*^*S*^ as follows. It generates (1) the *N*_*c*_ target words/phrases closest to *p*^*S*^ measured by the edit distance, and (2) *N*_*c*_ target words/phrases measured closest to *p*^*S*^ based on the cosine distance between their word-level embeddings in a bilingual space induced by the embedding model of Vulić and Moens [40]. As we will see in the experiments, besides straightforward gains in computational efficiency, limiting the number of candidates is even beneficial for the overall classification performance.

### Experimental setup

**Data** One of the main advantages of automatic BLI systems is their portability to different languages and domains. However, current standard BLI evaluation protocols still rely on general-domain data and test sets [8, inter alia; 38; 40; 57]. To tackle the lack of quality domain-specific data for training and evaluation of BLI models, we have constructed a new English-Dutch (EN-NL) text corpus in the *medical* domain. The corpus contains topic-aligned documents (i.e., for a given document in the source language, we provide a link to a document in the target language that has comparable content). The domain-specific document collection was constructed from the English-Dutch aligned Wikipedia corpus available online^7^, where we retain only document pairs with at least 40% of their Wikipedia categories classified as *medical*^8^. This simple selection heuristic ensures that the main topic of the corpus lies in the medical domain, yielding a final collection of 1198 training document pairs. Following standard practice [28, 45, 58], the corpus was then tokenized and lowercased, and words occurring less than five times were filtered out.

**Translation pairs: training, development, test** We constructed a set of EN-NL translation pairs using a semi-automatic process. We started by translating all words in our preprocessed corpus. These words were translated by Google Translate and then post-edited by fluent EN and NL speakers^9^. This yields a lexicon with mostly single word translations. In this work we are also interested in finding translations for phrases: therefore, we used IATE (Inter-Active Terminology for Europe), the EU’s inter-institutional terminology database, to create a gold standard of domain-specific terminology phrases in our corpus. More specifically, we matched all the IATE phrase terms that are annotated with the *Health* category label to the N-grams in our corpus. This gives a list of phrases in English and Dutch. For some terms a translation was already present in the IATE termbase: these translations were added to the lexicon. The remaining terms are again translated by resorting to Google Translate and post-editing.

We end up with 20,660 translation pairs. For 8,412 of these translation pairs (40.72%) both source and target words occur in our corpus^10^. We perform a 80/20 random split of the obtained subset of 8,412 translation pairs to construct a training and test set respectively. We make another 80/20 random split of the training set into training and validation data. 7.70% of the translation pairs have a phrase on both source and target side, 2.31% of the pairs consists of a single word and a phrase, 90.00% of the pairs consist of single words only. We note that 21.78% of the source words have more than one translation. In our corpus, the English phrases in the lexicon have an average frequency of 20. For Dutch phrases this is 17. English words in the lexicon have an average frequency of 59, for Dutch this number is 47.

**Word-level embeddings** Skip-gram word embeddings with negative sampling (SGNS) [34] are induced using the word2vec toolkit with the subsampling threshold set to 10*e*-4 and window size set to 5. BWESG embeddings [40] are learned by merging topic-aligned documents with length-ratio shuffling, and then training the SGNS model over the merged documents with the subsampling threshold set to 10*e*-4 and the window size set to 100. The dimensionality of all word-level embeddings in all experiments is *d*=50, and similar trends in results were observed with *d*=100.

**Classifier** The model is implemented in Python using Tensorflow [59]. For training we use the Adam optimizer with default values [60] and mini-batches of 10 examples. The number of negative samples 2*N*_*s*_ and candidate translation pairs during prediction 2*N*_*c*_ are tuned on the development set for all models except CHARPAIRS and CHARPAIRS -SGNS (see Experiments II, IV and V) for which we opted for default non-tuned values of 2*N*_*c*_=10 and 2*N*_*s*_=10^11^. The classification threshold *t* is tuned measuring *F*_1_ scores on the validation set using a grid search in the interval [0.1,1] in steps of 0.1.

**Evaluation metric** The metric we use is *F*_1_, the harmonic mean between recall and precision. While prior work typically proposes only one translation per source word and reports *Accuracy@*1 scores accordingly, here we also account for the fact that words can have multiple translations. We evaluate all models using two different modes: (1) *top* mode, as in prior work, identifies only one translation per source word (i.e., it is the target word with the highest classification score), (2) the *all* mode identifies as valid translation pairs all pairs for which the classification score exceeds the threshold *t*.

## Results and discussion

**A roadmap to experiments** We start by evaluating the phrase extraction (Experiment I) as it places an upper bound on the performance of the proposed system. Next, we report on the influence of the hyper-parameters 2*N*_*c*_ and 2*N*_*s*_ on the performance of the classifiers (Experiment II). We then study automatically extracted word-level and character-level representations for BLI separately (Experiment III and IV). For these single-component models Eq. 3 simplifies to $r_{h_{o}} = r^{ST}_{w}$ (word-level) and $r_{h_{o}} = r^{ST}_{c}$ (character-level). Following that, we investigate the synergistic model presented in the “Methods” section which combines word-level and character-level representations (Experiment V). We then analyze the influence on performance of: the number of hidden layers of the classifier, the training data size, and word frequency. We conclude this section with an experiment that verifies the usefulness of our approach for inducing translations with Greek/Latin roots.

### Experiment I: phrase extraction

The phrase extraction module puts an upper bound on the system’s performance as it determines which words and phrases are added to the vocabulary - translation pairs with a word or phrase that do not occur in the vocabulary can of course never be induced. To maximize the recall of words and phrases in the ground truth lexicon w.r.t. the vocabularies, we tune the threshold of the phrase extraction on our training set. The thresholds were set to 6 and 8 for English and Dutch respectively, and the value for *δ* was set to 5 for both English and Dutch. The resulting English vocabulary contains 13,264 words and 9081 phrases, the Dutch vocabulary contains 6417 words and 1773 phrases.

Table 1 shows the recall of the words and phrases in the training and test lexicons w.r.t. the extracted vocabularies. We see that the phrase extraction method obtains a good recall for translation pairs with phrases (around 80%) without hurting the recall of single word translation pairs^12^. The recall difference between English and Dutch phrase extraction can be explained by the difference in size of their respective corpora^13^.

**Table 1:** Recall of the words and phrases in the training and test lexicons w.r.t. the extracted vocabularies

	en		nl		en-nl	
	Phrases	Words+Phrases	Phrases	Words+Phrases	Phrases	Words+Phrases
Training lex.	86.26	97.03	72.06	95.31	80.96	99.51
Test lex.	88.60	97.12	67.44	95.62	79.69	99.11

In the EN-NL column we show the percentage of translation pairs for which both source and target words/phrases are present in the vocabulary. In the EN/NL columns we show the percentage of English/Dutch words/phrases that are present in the vocabulary

### Experiment II: hyper-parameters 2*N*_*c*_ and 2*N*_*s*_

**Fig. 4:**
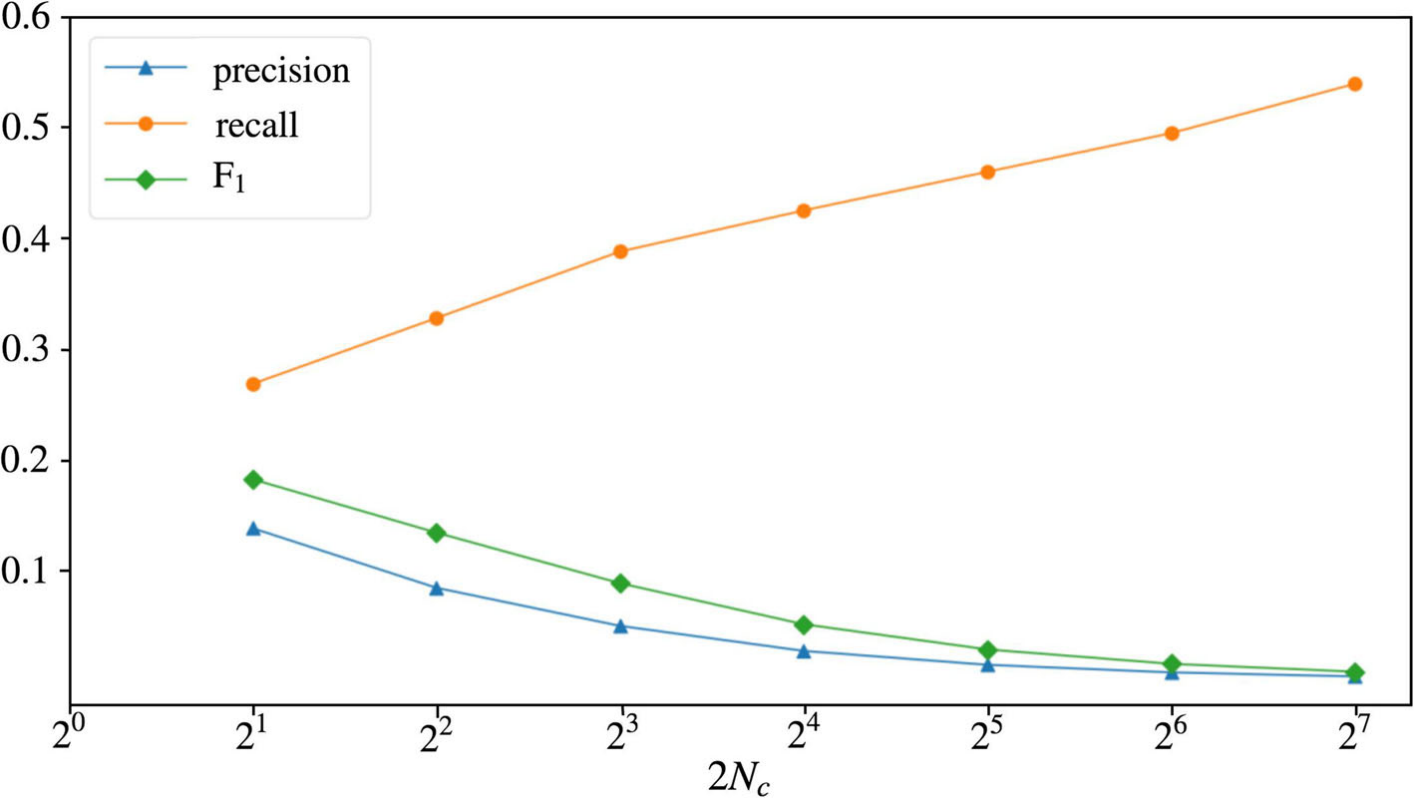
Precision, recall and F_1_ for candidate generation with 2*N*_*c*_ candidates

Figure 4 shows the relation between the number of candidates 2*N*_*c*_ and precision, recall and *F*_1_ of the candidate generation (without using a classifier). We see that the candidate generation works reasonably well with a small number of candidates and that the biggest gains in recall are seen when 2*N*_*c*_ is small (notice the log scale).

From the tuning experiments for Experiment III and IV we observed that using large values for 2*N*_*c*_ gives a higher recall, but that the best *F*_1_ scores are obtained using small values for 2*N*_*c*_; The best performance on the development set for the word-level models was obtained with 2*N*_*c*_=2 (Experiment III), for the character-level models this was with 2*N*_*c*_=4 (Experiment IV). The low optimal values for 2*N*_*c*_ can be explained by the strong similarity between the features that the candidate generation and the classifiers use respectively. Because of this close relationship, translations pairs that are lowly ranked in the list of candidates should also be difficult instances for the classifiers. Increasing the number of candidates will result in a higher number of false positives, which is not compensated by a sufficient increase of the recall.

We found that the value of 2*N*_*s*_ is less critical for performance. The optimal value depends on the representations used in the classifier and on the value used for 2*N*_*c*_.

### Experiment III: word level

In this experiment we verify if word embeddings can be used for BLI in a classification framework. We compare the results with the standard approach that computes cosine similarities between embeddings in a cross-lingual space. For SGNS-based embeddings, this cross-lingual space is constructed following [34]: a linear transformation between the two monolingual spaces is learned using the same set of training translation pairs that are used by our classification framework. For the BWESG-based embeddings, no additional transformation is required, as they are inherently cross-lingual. The neural network classifiers are trained for 150 epochs.

The results are reported in Table 2. The SIM header denotes the baselines models that score translation pairs based on cosine similarity in the cross-lingual embedding space; The CLASS header denotes the models that use the proposed classification framework.

**Table 2:** Comparison of word-level BLI systems

		**Development**					
		**Words**		**Phrases**		**Words + Phrases**	
**Representation**		*F*_1_ (top)	*F*_1_ (all)	*F*_1_ (top)	*F*_1_ (all)	*F*_1_ (top)	*F*_1_ (all)
sim	bwesg	13.48	9.15	21.95	15.84	14.24	9.73
	sgns	0.55	0.88	NaN	NaN	0.51	0.80
class	bwesg	17.08	21.19	24.04	26.47	17.59	21.56
	sgns	23.83	25.05	25.77	27.27	23.99	25.22
		**Test**					
		**Words**		**Phrases**		**Words + Phrases**	
**Representation**		*F*_1_ (top)	*F*_1_ (all)	*F*_1_ (top)	*F*_1_ (all)	*F*_1_ (top)	*F*_1_ (all)
sim	bwesg	12.78	10.03	21.43	12.52	13.52	10.31
	sgns	0.22	0.69	NaN	0.93	0.20	0.71
class	bwesg	16.47	21.50	23.48	23.75	17.01	21.68
	sgns	22.80	24.41	26.74	27.14	23.10	24.62

The best scores are indicated in bold

The results show that exploiting word embeddings in a classification framework has strong potential as the classification models significantly outperform the similarity-based approaches. The classification models yield best results in *all*-mode, this means they are good at translating words with multiple translations. For BWESG in the similarity-based approach, the inverse is true, it works better when only it proposes a single translation per source word.

We also find that the SGNS embeddings [34] yield extremely low results^14^. In this setup, where the embedding spaces are induced from small monolingual corpora and where the mapping is learned using infrequent translation pairs, the model seems unable to learn a decent linear mapping between the monolingual spaces. This is in line with the findings of [43].

We observe that in the classification framework SGNS embeddings outperform BWESG embeddings. This could be because SGNS embeddings can better represent features related to the local context of words such as syntax properties, as SGNS is typically trained with much smaller context windows compared to BWESG^15^. Another general trend we see is that word-level models are better in finding translations of phrases. This is explained by the observation that the meaning of phrases tends to be less ambiguous, which makes word-level representations a reliable source of evidence for identifying translations.

### Experiment IV: character level

This experiment investigates the potential of learning character-level representations from the translation pairs in the training set. We compare this approach to commonly-used, hand-crafted features. The following methods are evaluated:

- CHARPAIRS, uses the representation $r^{ST}_{c}$ of the character-level encoder as described in the “*Methods*” section and illustrated in Fig. 2.
- ed_*norm*_, uses the edit distance between the word/phrase pair divided by the average character length of *p*_*s*_ and *p*_*t*_, following prior work [44, 61].
- log(ed_*rank*_), uses the logarithm of the rank of *p*_*t*_ in a list sorted by the edit distance w.r.t. *p*_*s*_. For example, a pair for which *p*_*t*_ is the closest word/phrase in edit distance w.r.t. *p*_*s*_, will have a feature value of *log*(1)=0.
- ed_*norm*_ + log(ed_*rank*_), concatenates the ed_*norm*_ and log(ed_*rank*_) features.

The ED-based models comprise a neural network classifier similar to CHARPAIRS, though for ED_*norm*_ and log(ED_*rank*_) no hidden layers are used because the features are one-dimensional. For the ED-based models, the optimal values for the number of negative samples 2*N*_*s*_ and the number of generated translation candidates 2*N*_*c*_ were determined by performing a grid search, using the development set for evaluation. For the CHARPAIRS representation, the parameters 2*N*_*s*_ and 2*N*_*c*_ were set to the default values (10) without any additional fine-tuning, and the number of LSTM cells per layer was set to 512. We train the ED-based models for 25 epochs, the CHARPAIRS model takes more time to converge and is trained for 250 epochs.

The results are shown in Table 3. We observe that the performance of the character-level models is quite high w.r.t. the results of the word-level models in Experiment III. This supports our claim that character-level information is of crucial importance in this dataset and is explained by the high presence of medical terminology and expert abbreviations (e.g., *amynoglicosides, aphasics, nystagmus, EPO, EMDR* in the data; see also Fig. 1), which because of its etymological processes, often contain morphological regularities across languages. This further illustrates the need of fusion models that exploit both word-level and character-level features. Another important finding is that the CHARPAIRS model systematically outperforms the baselines, which use hand-crafted features, indicating that learning representations on the character level is advantageous. Unlike the word-level models, translation pairs with phrases have lower performance than translations with single words. This is to be expected as phrases usually consist of a longer character sequence and hence are more difficult to represent.

**Table 3:** Comparison of character-level BLI methods from prior work [44, 45] with automatically learned character-level representations

	**Development**					
	**Words**		**Phrases**		**Words + Phrases**	
**Representation**	*F*_1_ (top)	*F*_1_ (all)	*F*_1_ (top)	*F*_1_ (all)	*F*_1_ (top)	*F*_1_ (all)
ED _*norm*_	24.49	19.53	15.62	19.87	23.83	19.55
log(ED _*rank*_)	28.57	28.17	18.05	17.27	27.86	27.46
ed_*norm*_+ log(ED _*rank*_)	25.99	11.20	18.40	14.35	25.49	11.31
CHARPAIRS	31.95	32.32	23.70	25.97	31.39	31.92
	**Test**					
	**Words**		**Phrases**		**Words + Phrases**	
**Representation**	*F*_1_ (top)	*F*_1_ (all)	*F*_1_ (top)	*F*_1_ (all)	*F*_1_ (top)	*F*_1_ (all)
ED _*norm*_	28.10	28.29	8.70	8.63	26.97	27.24
log(ED _*rank*_)	29.30	28.95	19.48	19.35	28.70	28.39
ed_*norm*_+ log(ED _*rank*_)	29.76	29.65	17.57	17.45	29.05	29.00
CHARPAIRS	30.70	32.19	31.82	30.61	30.81	32.15

The best scores are indicated in bold

### Experiment V: combined model

On their own the single-component word-level and character-level BLI models already perform very well in the task of biomedical BLI. In this experiment, we report the results of the combined model. In this setup, the LSTM network has 256 memory cells in each layer^16^, and SGNS embeddings were selected as word-level representations. The embeddings are trained a priori, whereas the character-level representations are trained jointly with the rest of the network. This configuration will encourage the network to learn new character-level information which is distinctive from the word-level representations.

Table 4 shows the results of the combined model together with the best single component models. As hypothesized, we obtain the best results with the combined model. For phrases however, CHARPAIRS -SGNS’s performance is lower than the single component models. Our hypothesis for this behavior is that the LSTM in the combined model has less memory cells in the LSTM layers. We found that having 256 memory cells, rather than 512 cells as in the CHARPAIRS model, gives best results overall. However, for a combined model with 512 cells we get an improved performance for the phrases. Table 5 shows translations induced by the different models that illustrate the advantage of a hybrid model. We observe that the CHARPAIRS model has learned that the first characters of words/phrases are very informative, though this sometimes results in false positives. The SGNS model sometimes confuses words that are semantically related, e.g., *zwangerschap (pregnancy)* and *miskraam (miscarriage)*. The CHARPAIRS -SGNS model is able to filter out false positives by exploiting both representations simultaneously. Even in cases where both single component models predict the wrong translations, it is possible that the combined model induces the correct translation(s) (e.g., *injected-ingespoten*).

**Table 4:** Results of the model that combines word-level and character-level representations (CHARPAIRS -SGNS) and the best performing single component models (CHARPAIRS and SGNS)

	**Development**					
	**Words**		**Phrases**		**Words + Phrases**	
**Representation**	*F*_1_ (top)	*F*_1_ (all)	*F*_1_ (top)	*F*_1_ (all)	*F*_1_ (top)	*F*_1_ (all)
CHARPAIRS	31.95	32.32	23.70	25.97	31.39	31.92
sgns	23.83	26.36	17.37	17.08	25.77	25.81
CHARPAIRS -SGNS	34.57	33.61	18.18	23.29	33.47	32.99
	**Test**					
	**Words**		**Phrases**		**Words + Phrases**	
**Representation**	*F*_1_ (top)	*F*_1_ (all)	*F*_1_ (top)	*F*_1_ (all)	*F*_1_ (top)	*F*_1_ (all)
CHARPAIRS	30.70	32.19	31.82	30.61	30.81	32.15
sgns	22.80	24.41	26.74	27.14	23.10	24.62
CHARPAIRS -SGNS	34.34	34.60	23.17	26.59	33.60	34.15

The best scores are indicated in bold

**Table 5:** Predicted translations of single component models and the combined model, illustrating the advantage of the combined model. Correct translations are in bold

Source word	Predictions CHARPAIRS	Predictions sgns	Predictions CHARPAIRS -SGNS
Miscarriage	/	zwangerschap, **miskraam**, cardiale	**miskraam**
Contractions	contraststof	**samentrekkingen**	**samentrekkingen**
Injected	injecties, injectie	naald	**ingespoten**
Desensitization	**desensitisatie**	injecties, **desensibilisatie**, ventilation	**desensibilisatie**, **desensitisatie**
Hart attack	**hartinfarct**, **hartaanval**, hartmassage	**hartaanval**, atherosclerose, tia	**hartinfarct**, **hartaanval**
Multifocal	multiple, **multifocale**	dominante	**multifocale**

**Fig. 5:**
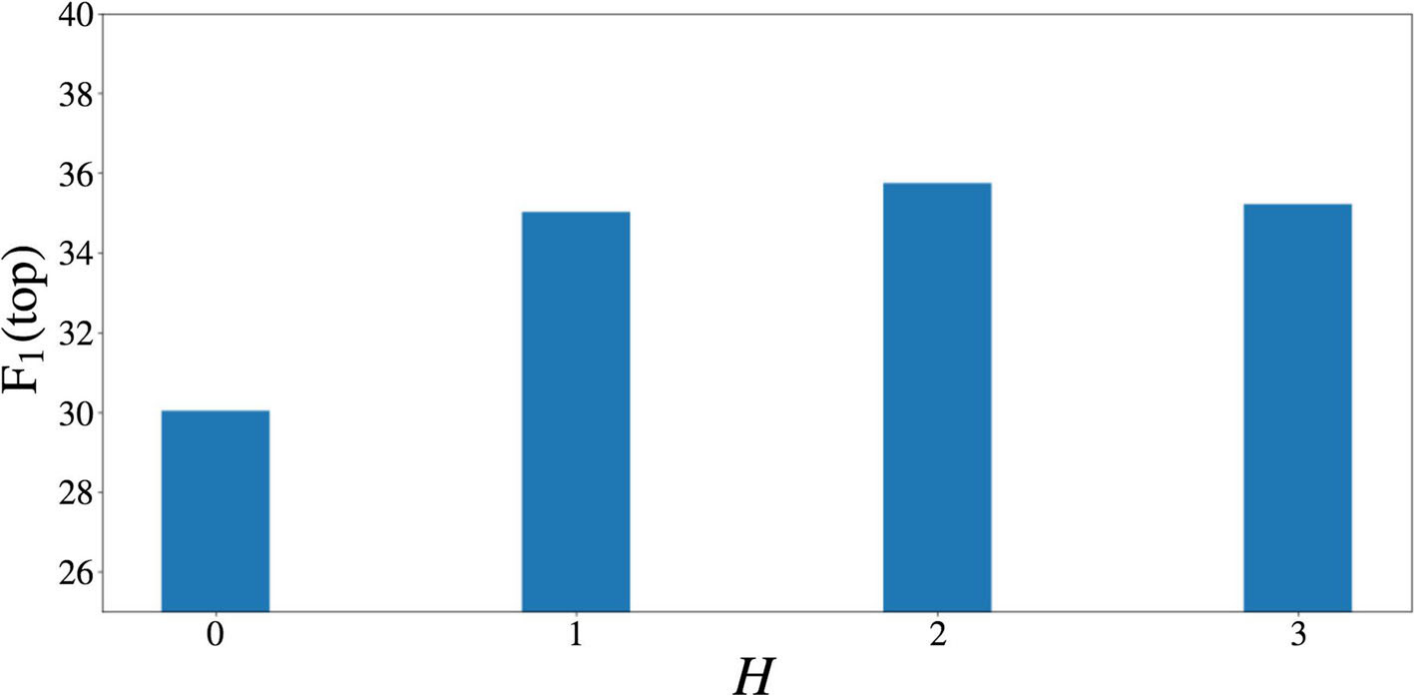
Hidden layers. The influence of the number of layers *H* between the representations and the output layer on the BLI performance

**Influence of the number of hidden layers*H*** The number of hidden layers *H* is a pertinent hyper-parameter. Figure 5 shows the influence of *H* on the performance measured by F_1_ in *top* mode. We see a large improvement when *H* ranges from 0 to 1. When there are no hidden layers (*H*=0), the network is unable to incorporate dependencies between features. In case the number of hidden layers is larger than one, we notice no large effect of the number of hidden layers on performance.

**Fig. 6:**
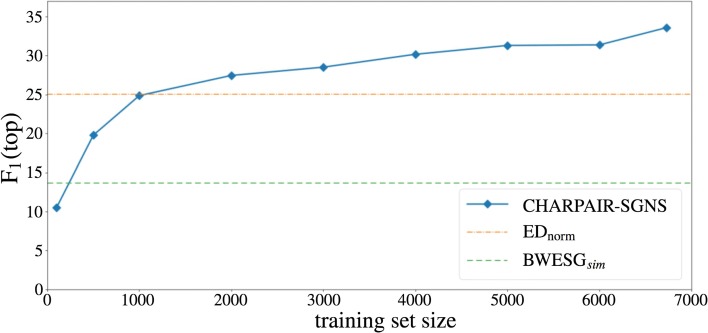
Training set size. The influence of the training set size (the number of training pairs)

**Influence of training set size** In many realistic settings, especially when dealing with languages and domains that have limited translation resources, we lack large numbers of readily available translation pairs. Figure 6 illustrates the influence of training set size on the performance of CHARPAIRS -SGNS. We also plot the performance of two of our baseline models that only use training data to tune the threshold *t*: BWESG embeddings combined with cosine similarity (see Table 2) and normalized edit distance (ED_*norm*_, see Table 3). We plot the performance of the baselines on the complete training set and assume it constant over the training examples. Unsurprisingly, the CHARPAIRS -SGNS performance increases with more training examples. Already from a seed lexicon size of 2000 translations it starts outperforming the baseline models.

**Fig. 7:**
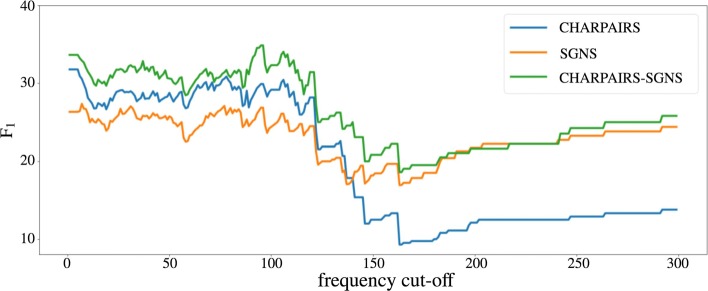
Word frequency. This plot shows how performance varies when we filter out translation pairs with frequency lower than the specified cut-off point (on x axis)

**Influence of frequency** In Fig. 7 we see the effect of word/phrase frequency on performance. We plot F_1_ scores after filtering the predicted translations and test set with a minimum word frequency cut-off. For example, for a cut-off frequency of 10, we only evaluate the translation pairs for which source and target words/phrases occur at least 10 times. Until a cut-off value of 125 performance for the three representations fluctuates but remains roughly level. When we only evaluate on high-frequency words (> 125) we see a performance drop for all models, especially for the character-level only model. From a manual inspection of these words we find that they typically have a broader meaning and are not particularly related to the medical domain (e.g., *consists-bestaat*, *according-volgens*, etc.). For these words, character-level information turns out to be less important.

**Translation pairs derived from Latin or Greek** We conclude the evaluation by verifying the hypothesis that our approach is particularly effective for translation pairs derived from Latin or Greek. Table 6 presents the F_1_ scores on a subset of the test data in which only translation pairs for which the English word or phrase has clear Greek or Latin roots are retained. The results reveal that character-level modeling is indeed successful for these type of translation pairs. All models scored significantly higher on this subset, surprisingly also the SGNS model. The higher scores of the SGNS model, which operates on the word-level, could be attributed to an increased performance of the candidate generation, as it uses both word- and character-level information. Regarding the differences between models, the same trends as in previous model comparisons are apparent: the CHARPAIRS model improves nearly 5% over the edit distance baseline and the CHARPAIRS -SGNS model achieves the best results.

**Table 6:** Results on a subset of the test data consisting of translation pairs with Greek or Latin origin

	ED _*norm*_	CHARPAIRS	SGNS	CHARPAIRS -SGNS
*F*_1_ (top)	50.25	54.46	42.92	57.20
*F*_1_ (all)	50.23	55.04	48.14	56.41

The best scores are indicated in bold

## Conclusions

We have proposed a neural network based classification architecture for automated bilingual lexicon induction (BLI) from biomedical texts. Our model comprises both a word-level and character-level component. The character-level encoder has the form of a two-layer long short-term memory network. On the word level, we have experimented with different types of representations. The resulting representations were used in a deep feed-forward neural network. The framework that we have proposed can induce bilingual lexicons which contain both single words and multi-word expressions. Our main findings are that (1) taking a deep learning approach to BLI where we learn representations on word-level and character-level is superior to relying on handcrafted representations like edit distance and (2) the combination of word- and character-level representations proved to be very successful for BLI in the biomedical domain because of a large number of orthographically similar words (e.g., words stemming from the same Greek or Latin roots).

The proposed classification model for BLI leaves room for integrating additional translation signals that might improve biomedical BLI such as representations learned from available biomedical data or knowledge bases.

## Abbreviations

BLI: Bilingual lexicon induction
BWE: Bilingual word embedding
BWESG: Bilingual word embedding skip-gram ED edit distance
LSTM: Long short-term memory
RNN: Recurrent neural network
SGNS: Continuous skip-gram with negative sampling

## Footnotes

1 For instance, UMLS currently spans only 21 languages, and only 1.82% of all terms are provided in French.

2 We refer to recent comparative studies [42, 43] for a thorough explanation and analysis of the differences between BWE models.

3 This paper expands research previously published in [51] by making the proposed model applicable to phrases and by adding more qualitative and quantitative experiments.

4 If we accidentally construct a pair which occurs in the set of positive pairs *D*_*lex*_, we re-sample until we obtain exactly *N*_*s*_ negative samples.

5 A possible modification to the architecture would be to swap the (unidirectional) LSTM for a bidirectional LSTM [55]. In preliminary experiments on the development set this did not yield improvements over the proposed architecture, we thus do not discuss it further.

6 We used the implementation of the gensim toolkit https://github.com/RaRe-Technologies/gensim [56].

7
http://linguatools.org/tools/corpora/

8
https://www.dropbox.com/s/hlewabraplb9p5n/medicine_en.txt?dl=0

9 In case the post-editor was unsure about the automatically acquired translation, he researched the source term on the web and corrected the translation if necessary.

10 Since we work with a comparable corpus in our experiments, not all translations of the English vocabulary words occur in the Dutch part of the corpus and vice versa.

11 It takes more time to train and hence tune the models with the character-LSTM.

12 Note that when a word is always extracted as part of a phrase then it would not occur separately in the vocabulary.

13 The English corpus consists of ≈1246k word occurrences, the Dutch corpus of ≈ 413k word occurrences.

14 The NaN values in Table 2 are caused by an absence of true positives.

15 Note that BWESG uses large window sizes by design.

16 We found that in the combined setting of using both word-level and character-level representations, it is beneficial to use a LSTM of smaller size than in the character-level only setting.
